# Can the need for soft tissue release in total knee replacement be predicted pre-operatively? A study based on surgical navigation

**DOI:** 10.1007/s00264-021-05263-3

**Published:** 2021-11-24

**Authors:** Daniel Hernandez-Vaquero, Alfonso Noriega-Fernandez, Sergio Roncero-Gonzalez, Gorka Luis Ruete-Gil, Jose Manuel Fernandez-Carreira

**Affiliations:** 1 Department of Orthopedic Surgery, School of Medicine, University of Oviedo, Julian Claveria, s/n, 33006 Oviedo, Spain; 2 Hospital San Agustin de Aviles, Aviles, Spain; 3 Hospital de Jarrio, Coaña, Spain

**Keywords:** Total knee replacement, Knee deformity, Limb alignment, Computer-assisted knee replacement, Gap balancing technique, Measured resection technique, Soft tissue release

## Abstract

**Introduction:**

In complex and deformed knees, soft tissue release (STR) is required to obtain symmetry in the femorotibial gap. The objective of this study was to attempt to predict the need for soft tissue release using surgical navigation in total knee replacement (TKR).

**Methods:**

Prospective and non-randomized study. One hundred thirty knees. At the start of navigation, an attempt was made to correct the femorotibial mechanical axis by applying force to the medial or lateral side of the knee (varus-valgus stress angle test). A gap balanced technique with computer-assisted surgery (CAS) was performed in all cases. The ligaments were tensioned, and using CAS visualization and control, progressive STR was performed in the medial or lateral side until a symmetry of the femorotibial gap was achieved.

**Results:**

Eighty-two patients had a varus axis ≥ 3° and 38 had a valgus axis (*P* < 0.001). STR was performed under navigation control in 38.5% of cases, lateral release (LR) in 12 cases, and medial release (MR) in 38 cases. After performing the varus-valgus stress angle test (VVSAT), the axis of 0° could be restored at some point during the manoeuvre in 28 cases. STR was required in 44.6% of varus cases and 27% of valgus cases (*P* = 0.05). A significant relationship was found between the previous deformity and the need for MR (*P* < 0.001) or LR (*P* = 0.001). STR was more common in male patients (*P* = 0.002) and as obesity increased.

**Conclusion:**

This study shows that pre-operative factors favouring the need to perform STR in a TKR implant can be defined.

## Introduction

The objective of the total knee replacement (TKR) is to achieve a well-aligned and well-balanced knee [1]. Creation of symmetric balanced flexion and extension gaps (difference less than 3 mm) is a surgical goal of a TKR, which often requires a soft tissue release (STR) at the time of implantation [2]. The need to incorporate this STR introduces a complexity factor in the surgical procedure, and no techniques were available that allow for pre-operative prediction of its suitability. If this could be previously known, it would serve as a guide for the surgeon who faces a TKR in cases with severe frontal deformity.

In the search for dynamic soft tissue assessment, application of force in extension using the varus-valgus stress angle test (VVSAT) [3] and confirmation of the correction obtained has been recommended. The limits of the maximum femorotibial angulation achieved in this manoeuvre could be related to the subsequent need for medial (MR) or lateral release (LR) to achieve a normal limb axis.

Additionally, it has been attempted to relate the preoperative deformity measured in a frontal long X-ray to the asymmetry of the gap in extension, and therefore to the need for STR in the surgical procedure [4]. However, it has not been studied whether this relationship is constant, regardless of body mass index (BMI), patient sex, and the degree and type of deformity. It would be very useful for the surgeon to know at the time of pre-operative planning whether the need for performing these soft tissue releases is predictable.

The surgical procedures required to obtain symmetry of the gap are well defined in the literature, but since standard surgery does not offer the possibility to verify its effectiveness, they are often arbitrary and dependent on the surgeon’s experience and subjective impression. Computer-assisted surgery (CAS) provides mechanical, anatomical, and kinematic alignment of the knee, as it dynamically assesses the axis of the limb throughout the range of motion of the knee [5]. In addition, measurement of the pre-implant gap and its correction can be adequately performed using CAS as it allows accurate medial–lateral gap control and flexion–extension balancing while assessing limb alignment [6]. A key advantage of this technical approach was that it allows immediate observation of the outcome of the different surgical gestures performed, modification of their amplitude, and correction of errors or anomalous situations [7].

The objectives of this study were as follows:

1. To determine if the preoperative axis of the limb obtained by forcing the knee (VVSAT) can be related with the need for MR or LR. If at any time during this manoeuvre a femorotibial axis of ± 3° is obtained, could this mean that STR would not be required?
2. To determine whether MR or LR is related to BMI, patient sex, or prior radiographic deformity, and whether release is more common in knees with varus or valgus deformity.

Our study was based on CAS findings but was aimed at helping the surgeon who performs TKR implantation using a conventional procedure. To sum up, it was intended to determine whether the possibility of correcting the deformity using varus-valgus stress manoeuvres will make STR unnecessary, and to determine if it was possible by a preoperative long X-ray to measure the asymmetry in the gap that the surgeon will encounter during surgery, and therefore, whether STR would be required. If our objectives were achieved, they would provide guidance to the surgeon, who could pre-operatively plan the best soft tissue treatment.

## Materials and methods

### Patient population

This study was a prospective, non-randomized study. The series consists of 130 cases. There were 86 female and 44 male patients, 13 cases were bilateral. The mean age was 71.08 (SD 9.53). All patients in the study were subjected to X-rays of the knee and frontal long X-ray of the limb in a standing position, with complete knee extension and including a metallic calliper of known diameter located close to the knee. From the PACS (Picture Archiving and Communication System) and using a computer program (Impax 6.3.1. 2813, Agfa Healthcare N.U. Montsel, Belgium), images were sent to the surgical planning software (Agfa Orthopaedics Tools v 2.06). This tool was used to first calculate the anatomical and mechanical axes of the femur and tibia, and then the anatomical and mechanical axes of the limb. Measurements were done by two of the authors, who had a wide experience with use of this planning system. Cases in which full knee extension could not be obtained were not included in this study. Varus angulation was considered as positive and valgus angulation as negative. Informed consent was obtained from all individual participants included in the study. Approval for this study was granted by the Regional Ethics Committee (PI12/01098).

### Surgical technique

All patients were operated on by the same surgical team, and all had the Apex TKR (Corin Group, Gloucestershire, UK) implanted. In all cases, a closed navigation system with no previous images was used, which employs kinematic analysis of the hip, knee, and ankle and anatomical mapping of the knee to construct a working model (Nanostation, Total Knee Surgetics, Praxim, S.A., La Tronche, France) [8]. At the start of navigation, and after the markers were placed in the femur and tibia, an attempt was made to correct the femorotibial mechanical axis by applying force to the medial or lateral side of the knee (VVSAT), depending on the previous varus or valgus situation. Both the angulation at rest and those obtained after the forced manoeuvre were recorded in the navigation system. The force applied was as much as possible and was always performed by the same two surgeons.

**Fig. 1:**
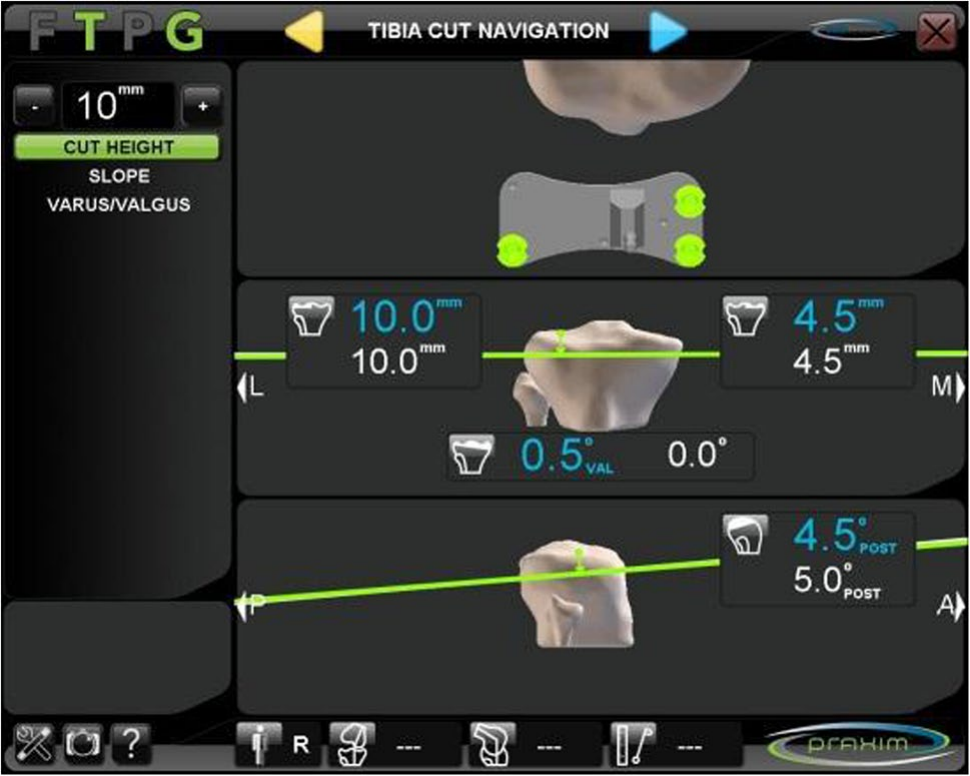
Tibial cut performed following the information provided by the navigation system

**Fig. 2:**
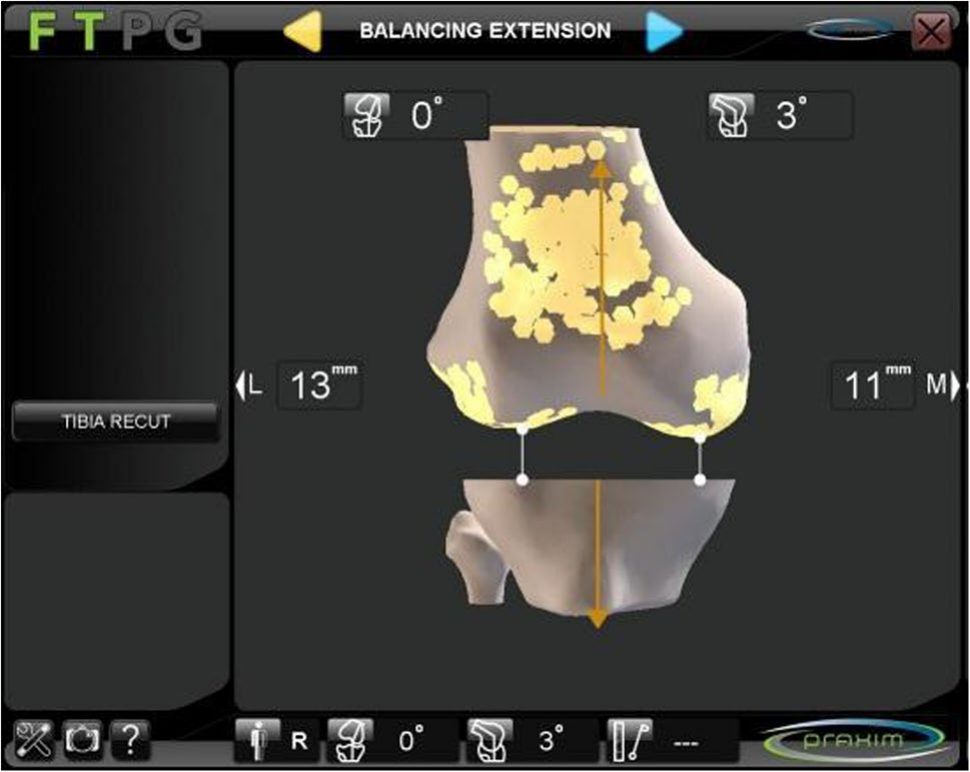
Verification of femorotibial gap in extension

**Fig.3:**
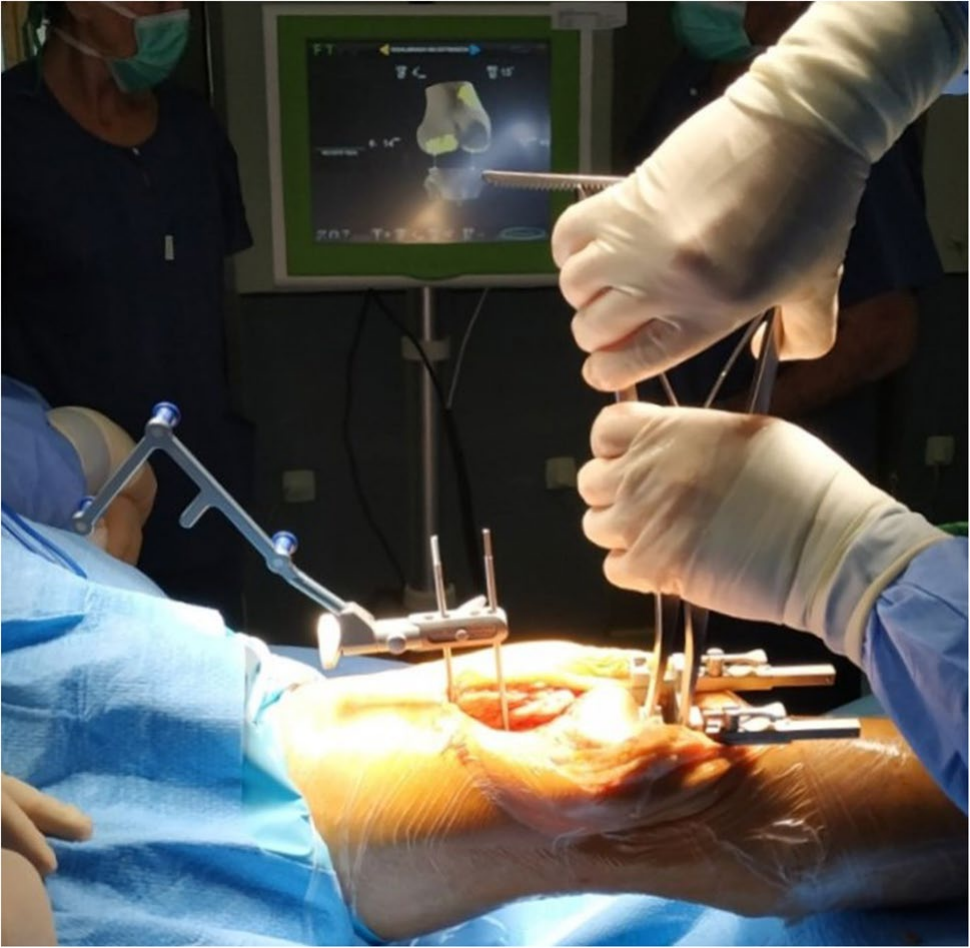
Medial and lateral femorotibial distraction with the tensioner, controlled by navigation

A gap balanced technique with CAS was performed in all cases. After removing the osteophytes, a tibial cut at 90° on the mechanical axis of the tibia was made in the coronal plane with 5° of posterior slope in the sagittal plane (Fig. 1); the initial femorotibial gap was recorded in extension (Fig. 2). Applying the dependent cut technique by sequential releases with a distractor, the femoral cut was calculated to obtain symmetric extension and flexion spaces with equal soft tissue tension. The ligaments were tensioned with an instrument that separates the tibia and femur, a so-called tensioner (Fig. 3); the measurement of the initial gap at rest and after maximal medial and lateral distraction was recorded on the navigation screen. The maximum distraction was performed by the two surgeons who performed all the interventions and the mean of both was used after progressive soft tissue release was performed until the medial and lateral space were equal [9]. The patella was restored to its anatomic position before the spaces were tightened. The obtained position was used to define the bone cuts of the femur and eventual position of the femoral component in all planes. The navigation system used records and stores all data by filing the information in an internal and exportable database.

### Statistics

All variables were studied descriptively. Adjustment of variables to normal was studied. Multiple regression analysis was performed for the dependent variable gap and the independent variables varus and valgus deformity and BMI. A multiple regression equation was obtained with the weight of independent variables as beta coefficients and their corresponding 95% confidence intervals. SPSS statistical packages for Windows version 20.0 and MedCalc version 9.3.1 were used.

## Results

### Patient characteristics

Mean BMI of the series was 31.17 (SD 5.55). Over half of patients (55.3%) were included in one the WHO-defined obesity categories. Cases with prior varus deformity were seen to have a significantly higher BMI than cases with valgus or neutral alignment (*P* = 0.013), but only a weak correlation was found between BMI and the degree of pre-operative deformity (Pearson’s correlation 0.143, *P* = 0.112). The pre-operative mechanical axis of the limb was a mean of 4.13° with oscillations between 27.4° varus and 26.0° valgus (SD: 11.93). Three groups were defined according to the pre-operative deformity of the mechanical axis measured in the long X-ray. Neutral axis was considered when angulation was 0° ± 2.9°, valgus deformity was less than − 3°, and varus deformity was greater than 3°. The largest group (83 patients) had a varus axis ≥ 3° and 37 had a valgus axis (*P* < 0.001). There were more males with varus or valgus deformity (*P* < 0.465).

### Relation between STR and pre-operative factors

**Fig. 4:**
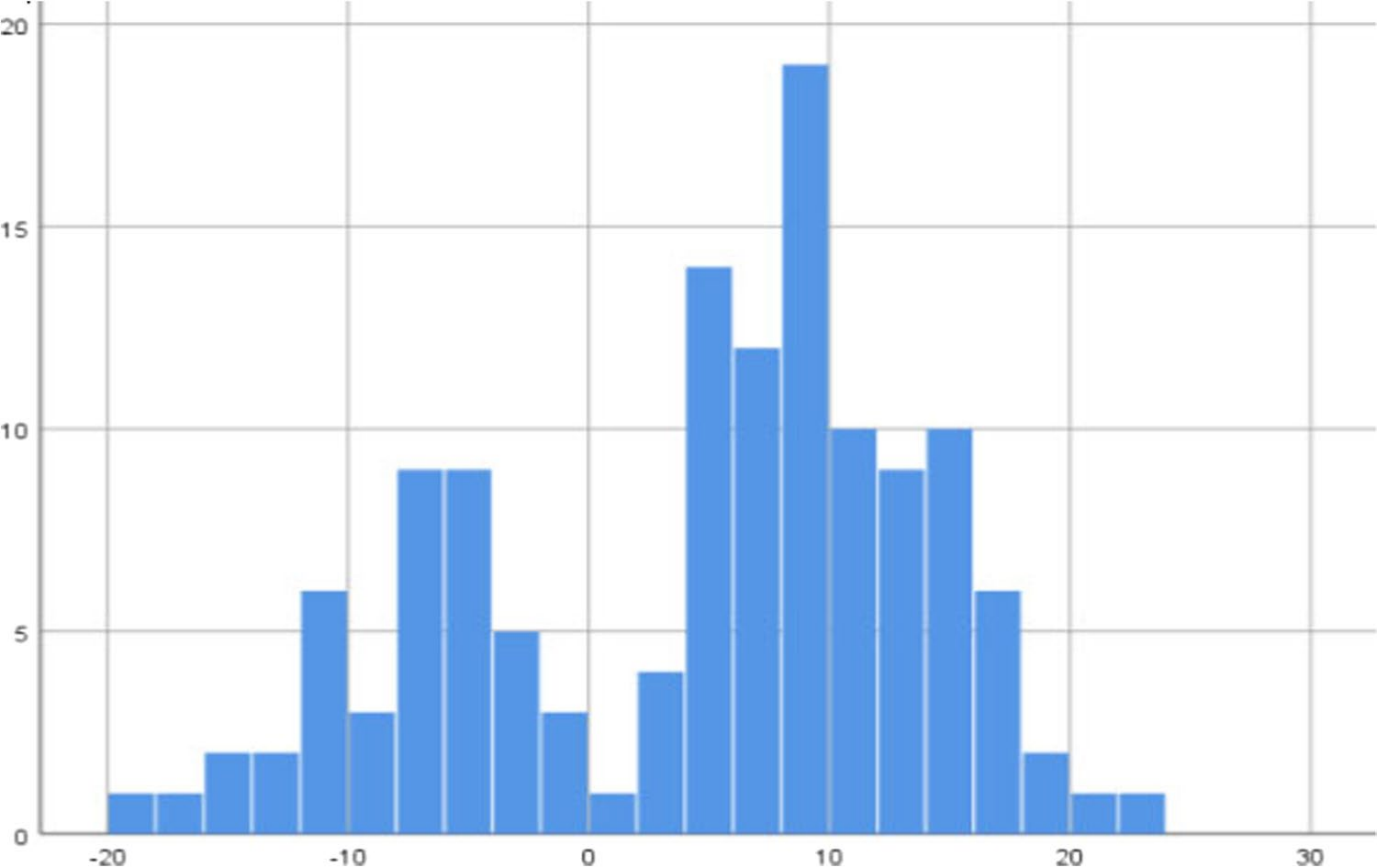
Femorotibial angle measured with CAS before surgery

After positioning the emitters in the femur and tibia and before starting the TKR implant, the initial femorotibial angulation provided by the navigation system was recorded. Mean limb axis was 4° (SD 9.41), ranging from 23° varus to 19° valgus. There were 38 knees with valgus deformity (mean 7.61°, SD 5.91) and 83 knees with varus deformity (mean 9.39°, SD 5.11) (Fig. 4), values close to the previously mentioned pre-operative radiographic measurement (37 and 83 respectively). After performing the VVSAT, 0° was achieved at some point during the flexo-extension manoeuvre in 28 cases, and if we consider the range of ± 3°, this was achieved in 82 cases. In cases where 0° was achieved at some point during the flexion–extension, release was performed in two cases and when 0° was not achieved in any moment, release was required in 48 cases (*P* < 0.001). When ± 3° post-VVSAT was achieved, release was performed in 19 cases and when angulation does not correct was achieved, release was required in 31 cases (*P* < 0.001) (Table 1). Achievement of a normal limb axis in the VVSAT suggests that LR or MR would not be required in the surgical procedure. In 50 cases (38.5%), STR was performed under control of the navigation system. This release was performed progressively until a symmetry was obtained between the medial and lateral gap. After this release, a lateral gap of 16.34 mm (SD 3.09) and a medial gap of 16.50 mm (SD 2.95) were obtained, with a difference of 0.08 (SD 3.09).

**Table 1:** STR when a neutral femoral-tibial axis was achieved in the VVSAT (*P* < 0.001)

Femoral-tibial angle	Achieved after VVSAT	STR	Not achieved after VVSAT	STR
0°	28	2	92	48
± 3°	82	19	38	31

*VVSAT*, varus-valgus stress angle test;

*STR*, soft tissue release

If we exclude out cases without radiological pre-operative deformity (10 cases), we find that 44.6% of varus cases and 27% of valgus cases required release (*P* = 0.05) (Table 2). There was a higher frequency of release in cases with greater deformities (*P* < 0.001). MR was performed in 38 cases (20 women and 18 men) and LR in 12 (9 women and 3 men). A significant relationship was found between the previous deformity and the need for MR (*P* < 0.001) or for LR (*P* = 0.001).

**Table 2:** STR and pre-operative deformity

		Not STR		STR		
		*n*	%	*n*	%	*n*
Pre-operative axis	Valgus	27	73.0%	10	27.0%	37
	Varus	46	55.4%	37	44.6%	83
Total		73	60.8%	47	39.2%	120

**Fig. 5:**
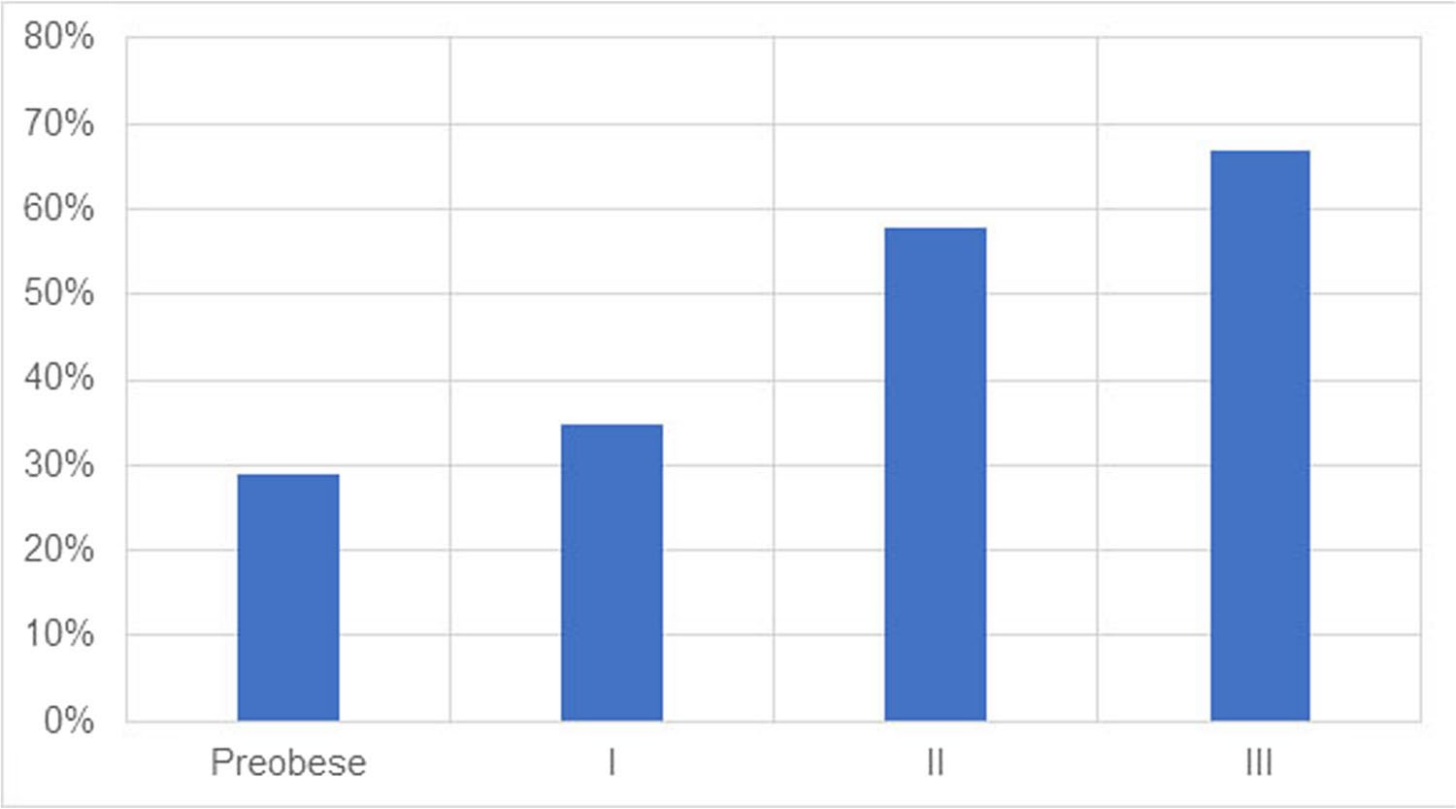
STR and the WHO obesity categories

Significant differences were found when MR was compared by patient sex, which was more common in males (*P* = 0.002). In patients with an elevated BMI, the need for release was more common (*P* = 0.079) with an increasing frequency as the degree of obesity increased. In almost 70% of cases with type III obesity, STR was required (Fig. 5).

## Discussion

Our work was based on data from the CAS and was aimed at surgeons who do not use this technique when implanting a TKR. In this regard, its main findings were, firstly, that if normalisation of the pre-operative axis of the limb was achieved after a varus-valgus forced manoeuvre, soft tissue release may not be required when TKR was implanted, and secondly, that from the pre-operative coronal mechanical axis of the limb and depending on deformity, sex, and BMI, the need for lateral or medial release may be predicted until symmetry of the femorotibial gap in extension was achieved.

To create a symmetrical gap, sequential medial and lateral releases were required depending on the deformity and previous ligament balance. There are two ways to perform the gap balancing technique: extension first or flexion first. Balancing in extension first, as was done in our study, was considered more reliable, as releases were more precise [10]. Although surgical procedures to achieve this should follow a systematised and progressive order [1, 11] STR increases the complexity of the procedure [12], so a correct alternative would be to do it only if necessary, knowing this could be helpful in pre-operative planning. A tool for objective quantification of the femorotibial space, resulting from limb alignment and the manoeuvre performed [13, 14] was CAS. The information provided by this technique may be useful for the surgeon performing the TKR implant using the conventional technique.

An attempt has been made to determine the factors influencing the reducibility of deformities by varus-valgus forced manoeuvres before TKR surgery. For some authors [11], the pre-operative varus deformity (measured by pre-operative mechanical axis angle) correlates well with reducibility of varus deformity. Mihalko and Krackow [15] suggested that a pre-operative distraction test and a varus­valgus stress test can provide useful information on medial and lateral ligament laxity. Other authors [16, 17] have noted in this same regard that a pre-operative distractive stress X-ray was useful for predicting the extent of medial release, and that if the mechanical axis of the limb was restored before bone cuts were made, no release would need to be performed. In none of the above studies was navigation used to check this relationship. CAS allows the modification of the limb axis to be known by performing the VVSAT in the knee before the start of surgery. It can check if a correct femorotibial axis was achieved and thus avoid soft tissue release. All this was done using a reproducible, reliable technique with data recorded before and after the forced manoeuvre. In our study, we observed by performing this manoeuvre with CAS that there was a relationship between the possibility of achieving a normal limb axis by VVSAT and the need to perform STR. It can be inferred from these findings that if the femorotibial axis was restored after VVAST, it is less likely that STR will be required in immediate surgery.

We found no studies like ours in the literature where the relationship between axis correction by VVSAT and the need to perform STR can be determined using CAS. Lee O-S et al. [3] performed the varus-valgus stress manoeuvre at a time prior to anaesthesia, which, from our point of view, cannot reproduce the true femorotibial laxity or rigidity or, therefore, the need for STR. In our series, VVSAT was performed after anaesthetic intervention and, therefore, without the bias that could be introduced by the presence of pain or muscle function.

Although the relationship between gap asymmetry and pre-operative X-rays has been partially studied [18], we also analysed whether the need for release until symmetry was achieved was related to patient sex, BMI, and prior deformity of the limb axis. For Matziolis et al. [4], the pre-operative axis of the limb was 9.8° on average, with limits of 20° valgus and 14.8° varus. In our series, the pre-operative axis was 4.13° on average, and had limits of 26.0° valgus and 27.4° varus. Possibly because of this frequency of cases with deformities, STR was performed in 38.5% of cases in our study. We analysed the need for release until a symmetrical gap in extension was achieved and found a significant relationship between prior deformity and the need for both medial and lateral release. There was a higher frequency of STR in cases with greater deformities. In almost half of the patients with varus deformity, STR was required, while it was performed in only 27% of the cases with valgus deformity.

BMI and sex also influenced the need for STR. Release was more common in males. In patients with high BMI, STR was more common, increasing in frequency as the degree of obesity increased, reaching 70% in patients with type III obesity. The variables sex and BMI were not evaluated in any study on STR and TKR deformities.

### Limitations

Our study has limitations. First, gap measurement after varus-valgus stress manoeuvres was performed manually. The final value was obtained by finding the mean of measurements taken by two different surgeons. Manual performance of this manoeuvre is widely referenced in the literature [3, 19–21] but we do not know whether the results would have been different if other techniques had been used. In our series, frontal deformities represent a high proportion of cases. We also do not know whether the results of this study would have changed if there had been a greater number of cases with a normal femorotibial axis. However, we think our study was justified by the difficulty offered by implanting a TKR in large deformities. Finally, we have only analysed the results obtained in knee extension, not in flexion. Our objectives were to relate frontal deformity measured with CAS and a long X-ray in extension to the need for STR.

## Conclusion

Our results, found after CAS use, may be useful in conventional surgery. From these findings, the group of patients who will require soft tissue release to obtain a normal femorotibial axis after TKR can be defined. If it is verified after applying the VVSAT that a normalisation of the axis was obtained, it can be predicted that release will not be necessary. The degree of pre-operative frontal radiographic deformity, patient sex, and BMI were also factors influencing the need to perform STR to obtain symmetry of the femorotibial gap.
